# Underwater Photogrammetry for the Study of Vulnerable Benthic Species: The Case of *Pinna rudis* Linnaeus, 1758

**DOI:** 10.3390/ani16121814

**Published:** 2026-06-12

**Authors:** Elena Prado, Luis Rodríguez-Cobo, Elvira Álvarez, Maite Vázquez-Luis

**Affiliations:** 1 Centro Oceanográfico de Santander, Instituto Español de Oceanografía (IEO, CSIC), 34004 Santander, Spain; elena.prado@ieo.csic.es; 2 Grupo de Ingeniería Fotónica, Department of Electronic Technology and Systems and Automation Engineering, Universidad de Cantabria, 39005 Santander, Spain; luis.rodriguez@unican.es; 3 Centro Oceanográfico de Baleares, Instituto Español de Oceanografía (IEO, CSIC), 07015 Palma de Mallorca, Spain; elvira.alvarez@ieo.csic.es

**Keywords:** SfM photogrammetry, marine protected species, marine vulnerable species, biodiversity conservation

## Abstract

The development of digital photogrammetry techniques has revolutionized the study of marine ecosystems, enabling the generation of high-precision three-dimensional models from conventional imagery. Structure from Motion (SfM) algorithms have become effective tools for mapping and monitoring underwater habitats, offering a non-invasive and cost-effective alternative to traditional methods. This study presents a pilot methodological validation of SfM-based underwater photogrammetry for the non-invasive morphometric monitoring of vulnerable benthic species, using *Pinna rudis*. The research focused on refining photogrammetric methodologies for marine conservation, addressing technical challenges such as variations in light conditions, water turbidity, and image acquisition complexity. The study area, the Cabrera Archipelago Maritime-Terrestrial National Park, is a pristine marine environment in the western Mediterranean, hosting diverse benthic communities, including an abundant *Pinna rudis* population. Data acquisition comprises sampling by scuba diving techniques at depths ranging from 26 to 31 m, performed during the July 2022 field campaign within a permanent demographic plot established in 2013 and the methodology applied involved generating three-dimensional models using SfM, allowing for direct measurements of the seabed and extraction of morphometric parameters of sessile species. The characterization of the *Pinna rudis* aggregation was based on specimen density and size structure, determined using maximum shell width. The 3D model of the pilot plot covers 86.1 m^2^, hosting 31 individuals. Morphometric measurements derived from SfM-based 3D models were validated against in situ diver measurements of maximum shell width. The results showed that the average maximum width obtained from 3D models (15.19 ± 3.23 cm) was consistent with in situ measurements (15.35 ± 3.48 cm). The mean difference between methods was −0.16 ± 0.82 cm, indicating a negligible systematic bias. The mean absolute error was 0.65 cm, corresponding to an average relative error of 4.34%, and a strong linear relationship was observed between both methods (r = 0.97). These results confirm that underwater photogrammetry is a reliable and non-invasive tool for monitoring vulnerable benthic species, providing high-resolution spatial and morphometric data to support conservation strategies in marine protected areas and allowing the collection of additional data compared to in situ surveys.

## Simple Summary

Monitoring marine species that live attached to the seabed is essential for conservation but remains challenging due to limited underwater working time and the risk of disturbing fragile organisms. Recent advances in image-based technologies now allow scientists to reconstruct highly detailed three-dimensional models of underwater environments from overlapping photographs, opening new possibilities for non-invasive monitoring. In this study, we applied and refined this approach to assess populations of the bivalve *Pinna rudis* in the Cabrera Archipelago Maritime-Terrestrial National Park. We developed a workflow capable of producing accurate three-dimensional representations of the seabed under complex conditions, including low light and confined spaces, and used these models to estimate individual size and population density. The results closely matched measurements obtained directly by divers, confirming the accuracy and reliability of the method. Beyond reducing field effort and disturbance, this technique enables the collection of detailed spatial information that is difficult to obtain using conventional surveys. By combining precision, efficiency, and minimal impact, this approach represents a significant step forward in the monitoring of vulnerable marine species and provides a powerful tool to support conservation and management in protected areas.

## 1. Introduction

Human activities and the effects of climate change have a significant impact on marine ecosystems, leading to habitat loss, resource overexploitation, the introduction of non-native and invasive species, and pollution. One of the most severe consequences of these impacts is biodiversity loss [1]. Various institutions are placing special emphasis on adopting new strategies to halt this biodiversity decline and restore ecosystems whenever possible. In this context, Marine Protected Areas (MPAs) play a crucial role. In fact, MPAs are a fundamental component of all national and international conservation strategies. Different administrative and environmental management frameworks, such as the Natura 2000 Network and National Parks, ensure the protection of these areas while also supporting research and scientific studies within them. However, effectively monitoring the species and habitats within these protected areas remains a major challenge, requiring precise, non-invasive, and scalable survey methods.

Recent advances in digital photogrammetry have profoundly transformed the way marine ecosystems are studied, allowing the reconstruction of high-precision three-dimensional (3D) models from standard photographic imagery. Among these methods, Structure from Motion (SfM hereafter) algorithms have emerged as powerful tools for the quantitative characterization of underwater benthic habitats and species. By combining principles of computer vision and spatial geometry, SfM enables the generation of metrically accurate 3D reconstructions from overlapping images, providing a non-invasive, flexible, and cost-effective alternative to traditional underwater monitoring techniques such as transect-based surveys or sonar mapping [2,3,4]. These technological advances have opened new possibilities for ecological research and conservation, particularly in environments where accessibility, fragility, or depth impose severe limitations on sampling effort. Additionally, underwater sampling using diving methodologies is often constrained by equipment or limitations in time that can be spent underwater, together with strict safety protocols [5,6].

The Cabrera Archipelago Maritime-Terrestrial National Park (CAMTNP), located in the Balearic Islands (western Mediterranean), constitutes one of the most pristine marine environments in the Mediterranean Sea. Its protection status has favoured the persistence of diverse benthic assemblages, including populations of the rough pen shell *Pinna rudis* [7]. This large benthic bivalve, belonging to the family Pinnidae, inhabits rocky substrates, coralligenous formations, sandy patches, and marine caves, where it plays a relevant ecological role by stabilizing sediments and providing structural complexity that benefits associated fauna [8,9,10]. Unlike its congeneric species *Pinna nobilis*, which has undergone a dramatic population collapse throughout the Mediterranean mainly by *Haplosporidium pinnae* epidemic [11,12], *P. rudis* appears to have maintained stable populations in certain areas, including Cabrera [13,14]. Its persistence makes it a suitable model species for the development of non-invasive monitoring approaches in Mediterranean benthic ecosystems. In addition, several morphological and ecological traits make *P. rudis* particularly well suited for photogrammetric monitoring: its sessile habit ensures that the same individuals can be repeatedly surveyed over time; its large shell size (up to 50 cm) and partially exposed posterior end provide unambiguous, well-defined landmarks for morphometric measurements; its high site fidelity and clumped distribution generate spatially structured aggregations amenable to discrete plot-based reconstruction; and its protected status under the Barcelona Convention favours non-invasive imaging methods over physical handling.

Its patchy distribution extends across the Mediterranean Sea and the eastern Atlantic Ocean, from the Gulf of Guinea to the Canary Islands and the Azores [15,16]. The species is typically found in shallow waters, ranging from 5 to approximately 70 m in depth [17], can grow up to 50 cm in shell length [18], and individuals are usually partially buried. Population densities tend to be low, ranging from 0.25 to 0.30 individuals per 100 m^2^ [19], even though most studies have been conducted in marine protected areas (MPAs) such as Scandola, Columbretes Islands, and Chafarinas. However, the highest density of *P. rudis* recorded to date within the CAMTNP reaches 6.89 individuals per 100 m^2^, documented specifically within the Foradada Siphon cave [7,20]. The very high density observed in this study reflects a targeted survey of the area with the highest aggregation of individuals and the intrinsically patchy distribution of the species. Several factors contribute to the unusually high local densities reported in CAMTNP relative to other Mediterranean MPAs. First, the National Park status of the archipelago, in force since 1991, has effectively excluded fishing, anchoring and other extractive pressures that affect *P. rudis* elsewhere. Second, the surveyed habitat—a semi-confined cave with sheltered hydrodynamic conditions, stable temperature regime and a coarse sand-and-gravel substrate interspersed with rocky microhabitats—appears to provide particularly favourable settlement and survival conditions for the species. Third, the local persistence of *P. rudis* in Cabrera contrasts with the regional decline of its congener *P. nobilis*, suggesting a possible release from interspecific competition, although this hypothesis requires further testing [12,13].

Despite its ecological relevance and apparent resilience, *P. rudis* remains insufficiently studied, although it is a catalogued species and included under the Barcelona Convention for the Protection of the Marine Environment and the Coastal Region of the Mediterranean. Quantitative data on its population structure, density, and habitat preferences are scarce, and long-term monitoring programs are absent [20]. Moreover, potential indirect impacts resulting from the decline of *P. nobilis*—through ecological interactions, changes in community composition, or shared habitat alterations—are largely unknown. Assessing the conservation status of *P. rudis* and understanding its spatial dynamics therefore requires the application of precise, repeatable, and minimally intrusive monitoring techniques capable of capturing fine-scale variation in both species’ morphology and habitat complexity [21]. Furthermore, these techniques are particularly relevant when working with vulnerable species in sensitive areas [22,23].

Underwater photogrammetry based on SfM algorithms represents a promising methodological framework for addressing these needs. The technique enables quantitative, high-resolution characterization of benthic species and habitats, providing centimetre-scale measurements of morphological and structural parameters such as shell width, height, exposed surface area, or substrate roughness. These metrics are essential for understanding population demography, growth patterns, and habitat associations, and can complement or replace traditional diver-based approaches that are often limited by time, cost, environmental conditions, and the number of measurements or variables that can be acquired in situ. Furthermore, SfM-derived models can serve as long-term spatial references for temporal comparisons, supporting monitoring programs aimed at detecting demographic or habitat changes over time [24,25].

Nevertheless, implementing photogrammetric techniques in underwater environments remains technically challenging. Light attenuation, turbidity, colour distortion, and platform instability can affect the quality of image alignment and the geometric accuracy of resulting models [26,27]. However, continuous improvements in camera calibration, image enhancement, and 3D reconstruction algorithms have significantly reduced these limitations, allowing for reliable data acquisition even in complex environments such as marine caves or low-visibility zones [6]. Therefore, in the present study, we present a pilot methodological validation of SfM-based underwater photogrammetry for the non-invasive monitoring of *Pinna rudis* in a marine protected area, focused on a high-density aggregation patch nested within a long-term permanent demographic plot. Specifically, we: (i) refine the methodological workflow for image acquisition and 3D reconstruction in confined and low-light underwater environments; (ii) validate the precision of morphometric measurements derived from 3D models against in situ observations; and (iii) evaluate the potential of this approach for the long-term, non-invasive monitoring of vulnerable benthic species.

## 2. Materials and Methods

The study was conducted within CAMTNP, a protected area located south-east of Mallorca in the Balearic Islands (western Mediterranean). The archipelago comprises 19 islets and covers approximately 90,800 ha, of which 89,482 ha are marine. The park encompasses a wide diversity of benthic habitats, including rocky reefs, coralligenous assemblages, seagrass meadows (*Posidonia oceanica* and *Cymodocea nodosa*), algal-dominated communities, and extensive submerged underwater cave systems. Specifically, this research is focused on the Foradada Siphon cave, situated off Illot de la Foradada on the northeastern side of Cabrera Island (approximate coordinates: 39°09′ N, 2°58′ E), at depths ranging from 26 to 31 m (for further details see [7]). The cave’s substrate is predominantly composed of coarse sand and gravel, interspersed with rocky sections that provide suitable microhabitats for *Pinna rudis*. This location was selected due to the presence of one of the highest reported densities of *P. rudis* in the Mediterranean [20], making it an ideal site to evaluate the performance of underwater photogrammetric methodologies for species monitoring. The sampling was carried out under strict compliance with the permits granted by the competent authorities: CEP 23/2022 and SEN 38/20-22.

### 2.1. Fieldwork and Data Acquisition

#### 2.1.1. In Situ Measurements

**Figure 1:**
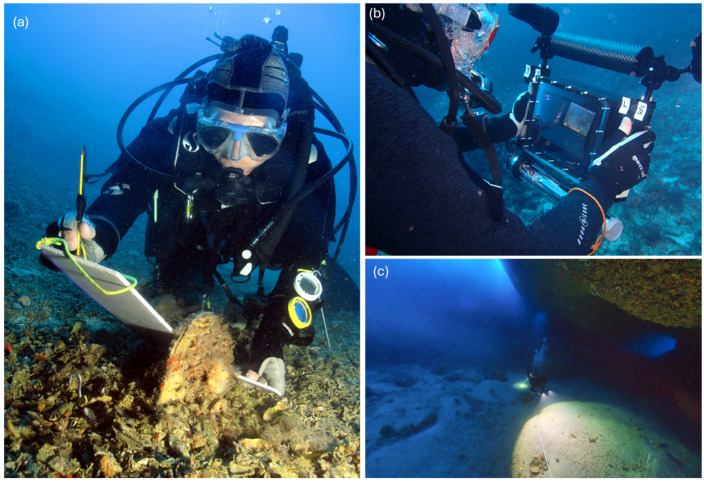
(**a**) Measuring the maximum shell width in situ using an L-shaped ruler. (**b**) Photogrammetry module designed for this study, with a Blackmagic Pocket Cinema 4K digital camera as a main component. (**c**) Scientific diver recording pre-defined video transects inside the Foradada Siphon cave.

Demographic monitoring requires a minimum number of individuals per plot, and it involves the monitoring of the same individuals over several years. The study of the demography of *P. rudis* presents constraints such as the often-fragmented distribution of the species, which makes it difficult to locate specimens, and the time that can be spent at a certain depth. In this study, sampling was performed in a demographic plot whose baseline was established in 2013 (marked and mapped), which has been visited annually. The permanent plot consists of a surface ~300 m^2^ in which there are more than 50 tagged *P. rudis* in a depth range from 27 to 35.6 m depth. The fieldwork of the present study was performed in July 2022. For demographic sampling, the individuals present in the plot were counted and mapped each year to check the survival of initially marked individuals. Unmarked individuals (recruits) were searched for and, if found, were mapped and marked for follow-up. Each individual in the plot was measured for maximum width underwater using an L-shaped ruler with a precision of ±1 mm [28], in order to assess the size structure of the population (Figure 1a). Moreover, the status of each individual was recorded (alive, dead). This type of monitoring provided the annual demographic rates of the population [29].

#### 2.1.2. Image Acquisition

In the same field survey, July 2022, systematic video transects were designed and performed for photogrammetric reconstruction. All dives were conducted following standardized image acquisition protocols to ensure proper spatial overlap, illumination uniformity, and camera stability throughout the surveys. The image acquisition focused on a part of the plot where there was a high density of specimens.

Video acquisition consisted of a single continuous recording of approximately 12 min, performed without interruption along parallel transects following a lawnmower pattern, with smooth turns at each end. The diver maintained a constant forward speed as low as practicable, a camera-to-substrate distance of approximately 1.0–1.5 m, and a fixed nadir-facing camera orientation throughout the survey. These parameters were controlled manually by a single diver without external assistance or guidance systems, which represents an inherent limitation in terms of precision of height and speed control, but also a key practical advantage: the acquisition protocol requires no additional equipment beyond the photogrammetry module itself and is fully compatible with standard scientific diving practice in shallow-water environments. Image overlap was ensured by the combination of low swim speed and a short camera-to-substrate distance, consistently exceeding 80% along-track and across-track. Water visibility within the Foradada Siphon cave during the survey was excellent, with no suspended particulate matter or turbidity detected in the video footage, and the short working distance between the camera and substrate (~1 m) further minimised any potential effect of light attenuation on image quality.

##### Custom-Built Photogrammetry Module

To overcome the limitations of conventional underwater cameras in low-light and confined environments such as marine caves, a custom photogrammetry module was designed, engineered, and constructed for this study [30]. The system was conceived to combine high optical performance, mechanical robustness, and integration flexibility for both diver-operated and remotely operated vehicle (ROV) missions (Figure 1b). The main component of the module is a Blackmagic Pocket Cinema 4K digital camera (Blackmagic Design, San Francisco, CA, USA), featuring a Micro Four Thirds sensor with exceptional low-light sensitivity and high dynamic range, ideal for capturing high-detail imagery under reduced illumination. The optical configuration includes a 12 mm f/2.8 prime lens, chosen for its balance between wide field of view, brightness, and compactness, enabling effective image coverage while minimizing optical distortion. The camera and control systems are enclosed within a custom-machined POM (polyoxymethylene) housing, specifically designed to withstand pressures corresponding to depths of up to 150 m. The optical interface consists of a commercial 5″ acrylic dome port (Hugyfot WAP 5″; Hugyfot, Aalst, Belgium), which ensures minimal refraction and high image clarity, while defining the maximum operational depth of the system. Illumination is provided by two high-intensity LED spotlights (CreeLED, Inc., Durham, NC, USA), positioned symmetrically on both sides of the optical axis to achieve even lighting and avoid shadowing on irregular surfaces. The lights emit a broad-spectrum, cool-white beam optimized for colour fidelity in underwater conditions. To ensure precise metric calibration during post-processing, the module incorporates two parallel laser pointers projecting fixed-distance beams onto the substrate, providing accurate scale references directly within each frame.

##### Electronic Architecture and Control Systems

The module’s electronic architecture integrates both local and remote-control systems to maximize operational versatility. A virtual interface module connects to the camera via Bluetooth, enabling remote adjustment of exposure, focus, ISO, and recording functions without direct physical contact. This interface also includes an RS232 communication port, allowing the system to be connected to external platforms such as small ROVs or auxiliary environmental sensors. This modular design facilitates integration into broader ecological monitoring frameworks, enhancing adaptability for future applications. Both the camera and the virtual interface are managed by ESP32 microcontrollers and powered by lithium polymer (LiPo) batteries, providing approximately two hours of autonomous operation. Power monitoring and thermal management were incorporated to maintain stable performance during prolonged dives.

To enable diver operation without compromising manoeuvrability, a dedicated underwater remote control unit was developed. This auxiliary controller is housed in a watertight tubular casing connected to the main module via an umbilical cable. It includes two magnetic switch panels linked to an ESP32-based microcontroller, which relay control signals to the main housing. The remote unit also contains independent batteries powering both the control electronics and communication systems. This configuration allows divers to start and stop video recording, adjust lighting intensity, and activate calibration lasers without physical manipulation of the main housing, thereby reducing diver movement and potential image instability.

##### Operational Deployment

During each dive, the photogrammetry module was manually operated by a diver swimming along pre-defined transects within the Foradada Siphon cave (Figure 1c). The camera was maintained at a distance of approximately 1.0–1.5 m from the substrate and moved at a constant speed to ensure >80% image overlap. The LED lights and calibration lasers remained continuously active to guarantee consistent illumination and scaling across frames. The specimen identification labels (nominal length: 0.041 m) affixed to each *P. rudis* individual remained visible throughout the transect and served as the sole metric scaling reference for the 3D model. The entire module was designed to achieve neutral buoyancy, minimizing diver effort and motion-induced blur while enhancing stability in confined environments. All system components were tested in shallow-water conditions prior to field deployment to verify optical alignment, housing integrity, and synchronization between the imaging and control subsystems. This configuration integrates cinema-grade image acquisition capability with the robustness and modularity required for scientific research.

### 2.2. Data Analyses

To assess the accuracy of SfM-based photogrammetry as an alternative to classical scientific diving surveys for *P. rudis* population monitoring, morphometric measurements derived from 3D photogrammetric models (maximum shell width, Wmax) were compared against in situ measurements of the same individuals obtained by scientific divers using a standardised protocol.

#### 2.2.1. Photogrammetric Processing and 3D Model Generation

Photogrammetry techniques based on SfM rely on the identification of homologous points in overlapping photographs or frames acquired through the movement of a single camera. Through automatic triangulation, this point correlation generates a three-dimensional point cloud that defines the terrain and determines the position and orientation of the camera along the recorded video transect. Subsequently, using computer vision principles, the three-dimensional point cloud is further densified to represent the terrain at a very fine scale, enabling the creation of derived products such as digital surface models and orthomosaics.

Video data were processed using PIX4Dmapper Pro v4.9.0 (Pix4D SA, Lausanne, Switzerland) on an Intel Core i7-11800H/NVIDIA RTX 3060 workstation. Frames were extracted directly from the video within the same software at a rate of one frame per 15 video frames (video recorded at 24 fps). Alternatively, dedicated video processing tools such as FFmpeg or DaVinci Resolve can be used for frame extraction prior to import into photogrammetric software. Initial processing used full-scale keypoint extraction with geometrically verified free-flight/terrestrial image matching, and camera self-calibration with full optimisation of internal and external parameters. No additional manual filtering was applied; frames that could not be calibrated or integrated into the adjustment model were automatically discarded by the software during the initial processing step. Dense point cloud generation was performed at the original image scale with High point density and a minimum of three matches per point. Approximate processing time for full 3D model generation was ~15 min on the described workstation, though this is highly dependent on hardware specifications.

Metric scaling was achieved exclusively using the specimen identification labels affixed to each *Pinna rudis* individual (nominal length: 0.041 m). Four scale constraints were defined as pairs of manual tie points on spatially distributed labels across the reconstruction area. The calibration laser pointers were not used as scaling inputs in this study. Although designed to provide in-frame scale references, the continuous camera displacement during acquisition and the low textural complexity of the homogeneous gravel substrate made it impractical to reliably identify the same projected points across successive frames in the 3D reconstruction workflow. Specimen identification labels, being geometrically discrete and visually unambiguous, provided a more robust and reproducible scaling solution.

This method generates a three-dimensional model that allows direct measurements of the seabed in three dimensions, enabling the extraction of morphometric parameters such as the height, volume, and roughness of the specimens.

#### 2.2.2. Morphometric and Population Characterization of *Pinna rudis*

The population of *P. rudis* in the study area was characterized through quantitative analyses derived from 3D photogrammetric reconstructions, supported by in situ diver-based measurements. Population metrics included individual density, size structure, and morphometric parameters describing shell shape and exposure. The size structure of the population was determined using the maximum shell width (Wmax) as the primary metric, which serves as a reliable indicator of age and growth stage in Pinnidae [31]. Additionally, the maximum exposed height (Hexp) was also quantified to provide a more comprehensive morphometric profile of each individual. These measurements were extracted directly from the 3D models generated using the Structure from Motion (SfM) workflow, allowing precise, non-invasive quantification of specimen dimensions. Measurements were obtained as follows: (i) maximum shell width (Wmax), defined as the straight-line distance between the two widest visible points of the shell valves and (ii) exposed height (Hexp), measured from the apex of the visible shell to the sediment surface, representing the degree of burial. These morphometric variables were measured for all identified individuals encountered within the reconstructed area, to characterize overall size distribution and population structure.

Morphometric measurements were extracted from the 3D point cloud using the manual measurement tool in PIX4Dmapper Pro. For each individual, a 3D line was initialised between approximate endpoints in the point cloud viewer and then refined by manually selecting the precise anatomical positions in each video frame where they were clearly visible (typically ~30 frames per measurement). PIX4Dmapper then automatically recomputed the optimal 3D distance, integrating all frame-level localisations, reducing the contribution of single-frame parallax to the final measurement. The estimated post-processing time was approximately 10 min per distance, yielding ~10 h total for the 62 measurements extracted (2 per individual × 31 individuals).

All measurements were performed by a single operator experienced in 3D point cloud analysis, following anatomical landmark definitions established in coordination with researchers experienced in in situ morphometric surveys of *Pinna rudis*. Formal intra- and inter-observer repeatability was not assessed, which represents a limitation of this pilot study. However, operator-dependent variability is similarly present in in situ caliper-based measurements, and the precision of the multi-frame refinement workflow is primarily determined by the geometric accuracy of the photogrammetric model. Repeatability assessment should be incorporated as this methodology is scaled up.

#### 2.2.3. Validation and Error Analysis

To assess the accuracy of photogrammetric measurements, the maximum shell width (Wmax) derived from 3D models was compared with in situ measurements obtained by scientific scuba divers. Measurement error was defined as the difference between both methods (W3D − Win situ). Accuracy was evaluated using mean error (bias), standard deviation of the error, mean absolute error (MAE), and relative error. Agreement between methods was further assessed using Pearson correlation and Bland–Altman analysis. Normality of differences was assessed using the Shapiro–Wilk test. Agreement between methods was evaluated using Pearson correlation, Bland–Altman analysis, and linear regression of 3D-derived versus in situ measurements. Systematic bias was tested using the Wilcoxon signed-rank test. The sample size (*n* = 31) corresponded to the totality of *P. rudis* individuals identified within the reconstructed high-density patch and is consistent with sample sizes reported in comparable photogrammetric validation studies of benthic invertebrates [22,23,25]. 

## 3. Results

### 3.1. Three-Dimensional Model Results

**Figure 2:**
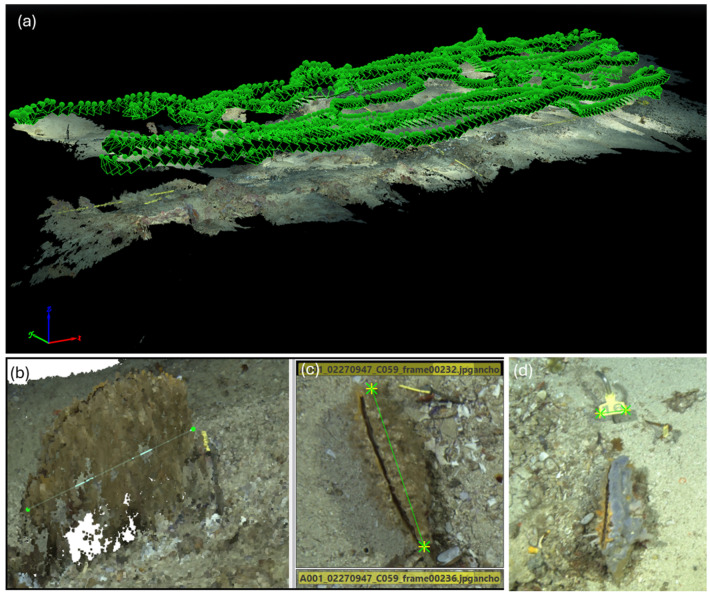
(**a**) The three-dimensional point cloud generated through SfM approach for the area where the target species were located, green squares represent the frames positions (**b**) Maximum shell width (Wmax) was extracted directly from the 3D model, and is defined as the straight-line distance between the two widest visible points of the shell valves. (**c**) The corresponding 2D images can be used to accurately adjust the maximum width (indicated with yellow and green asterisk). (**d**) Example of manual tie points (MTPs) and known distance constraints visible in the images, using the size of the labels used to identify specimens of *Pinna rudis*.

The three-dimensional reconstruction of the study area was generated using SfM photogrammetry in Pix4Dmapper v4.9.0, based on a dataset of 1217 underwater images acquired during the 2022 field campaign. Of these, 1209 images (99%) were successfully calibrated, evidencing a highly consistent and well-overlapping image network. The resulting model exhibited excellent internal geometry, high point density, and subcentimeter precision suitable for ecological and morphometric analysis (Table 1) (Figure 2a).

#### 3.1.1. Model Calibration and Accuracy

The bundle block adjustment stage yielded a total of 37,732,055 keypoint observations and 10,068,786 reconstructed 3D points, with a mean reprojection error of 0.477 pixels—well within the precision range expected for high-quality underwater reconstructions. The internal camera optimization produced a 3.02% relative difference between the initial and optimized intrinsic parameters, confirming minimal lens distortion and reliable calibration of the optical system. Each image contained a median of 48,507 keypoints, with an average of 33,111 matched features per calibrated frame, ensuring robust image connectivity and geometric redundancy. The overlap analysis indicated that most pixels were visible in at least five images, meeting the conditions for optimal reconstruction quality. These results confirm that the dataset achieved sufficient spatial redundancy to ensure high accuracy and model completeness, even in low-light and low-contrast zones typical of underwater cave environments.

#### 3.1.2. Scale and Geometric Constraints

Because no geolocation metadata were embedded in the image set, metric scaling was achieved using manual tie points and known distance constraints visible in the images, using the size of the labels used to identify specimens of *Pinna rudis* (Figure 2b–d). Four independent scale bars of 0.041 ± 0.001 m were used. A total of 28 manual tie points were distributed across the reconstruction to enhance local precision, with projection errors ranging from 1.27 to 3.25 pixels. This is consistent with the expected tolerance for underwater photogrammetric surveys. The combination of these constraints yielded a spatially stable model, allowing quantitative analyses with subcentimeter accuracy.

#### 3.1.3. Point Cloud Densification and 3D Mesh Generation

**Figure 3:**
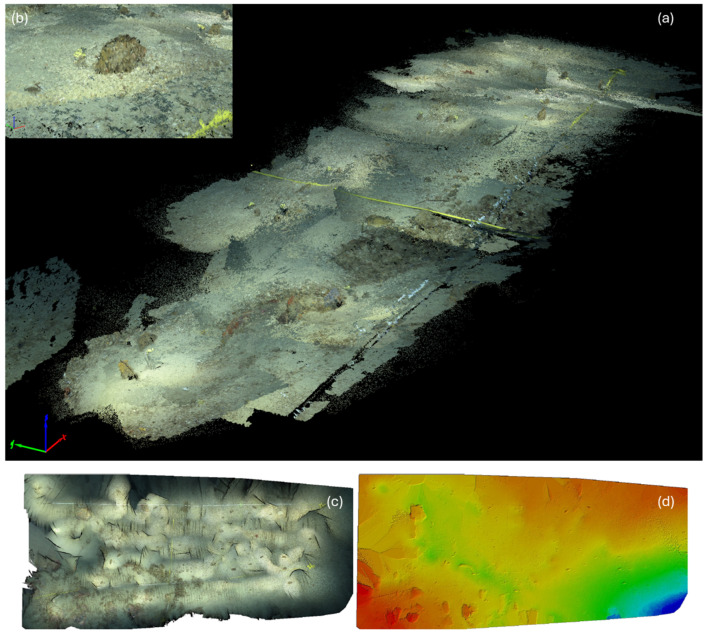
Three-dimensional point cloud, global view (**a**) and detail of a *Pinna rudis* specimen (**b**), orthomosaic (**c**) and the corresponding Digital Surface Model (DSM) of the study area (**d**). DSM colour scale represents relative elevation within the reconstructed model, not absolute georeferenced depth.

The dense point cloud was generated at full image resolution using multiscale processing and a high-density setting, requiring a minimum of three overlapping images per 3D point (Figure 3a). This produced 367,801,416 points, distributed across 23 tiles and covering approximately 86.1 m^2^ of the cave floor. The resulting model captured the fine-scale substrate complexity, including small sediment undulations, shell curvature, and partial burial of *Pinna rudis* specimens (Figure 3b). The 3D textured mesh, created at high-resolution settings, accurately reproduced both topographic detail and chromatic gradients, preserving the natural appearance of the substrate and organisms under artificial illumination. The absence of post-processing colour balancing was intentional, as it maintained the authentic tonal variation observed under the cave’s lighting conditions.

#### 3.1.4. Model Quality Assessment

The 3D model reconstruction exhibited strong photogrammetric geometry and high internal consistency. The optimized focal length and principal point coordinates closely matched the theoretical specifications of the imaging system, confirming the validity of the optical consistency. The combination of dense point sampling, low reprojection error, and precise scaling ensures that the model can be used for quantitative morphometric analyses of *Pinna rudis*, as well as for habitat mapping and structural complexity assessments. These attributes establish the model as a robust spatial baseline for long-term ecological monitoring in marine protected areas such as the Cabrera Archipelago National Park.

### 3.2. Population Description of Pinna rudis

**Figure 4:**
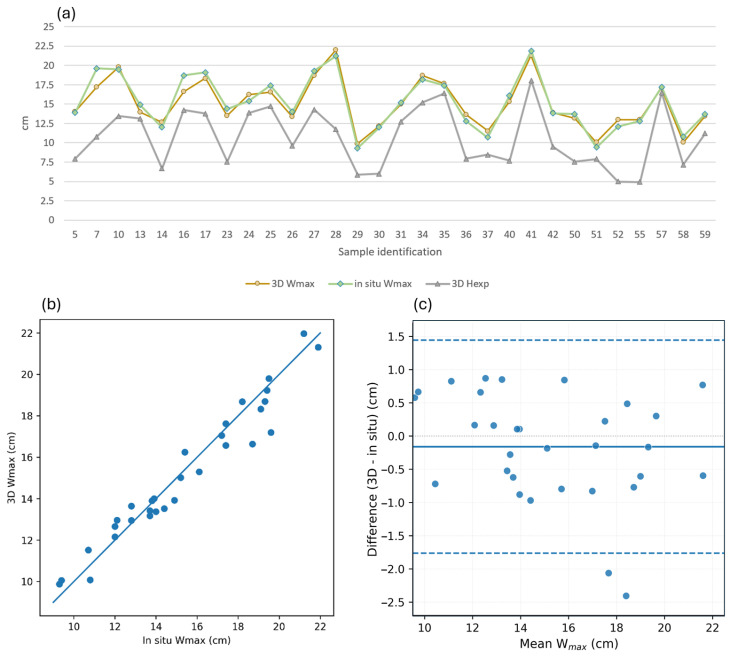
(**a**) Maximum shell width (Wmax) and Height exposed (Hexp) of *Pinna rudis* individuals measured from 3D photogrammetric models and in situ Wmax by scuba divers. Each point represents an individual specimen identified within the study area. (**b**) Direct comparison between 3D vs. in situ measurements, blue line indicate the adjustment. (**c**) Bland–Altman plot of 3D-derived vs. in situ Wmax. Solid line: mean difference (bias); dashed lines: 95% limits of agreement (±1.96 SD). Bias offset from zero reflects a small, non-significant systematic underestimation.

The results presented below describe the *P. rudis* aggregation observed within the photogrammetric reconstruction area (86.1 m^2^), which constitutes a high-density patch nested within the larger permanent demographic plot (~300 m^2^). The density and size-structure values reported in this section therefore characterise this specific high-density patch, used here for the methodological validation of the photogrammetric workflow, and should not be extrapolated to the wider permanent plot, the entire Foradada Siphon cave, or the broader *P. rudis* population in the Cabrera Archipelago. The three-dimensional model, orthomosaic and DSM generated for the study area cover a total surface of 86.1 m^2^ (Figure 3c,d). Within this reconstructed sector of the Sifón Cave floor, 31 individuals of *Pinna rudis* were identified, resulting in a density of approximately 36 individuals per 100 m^2^. All 31 specimens detected within the reconstructed area were alive at the time of the survey; no dead individuals or empty shells were identified. Therefore, all density and size-structure metrics reported here refer exclusively to live individuals. It is important to emphasize that the modelled area represents the zone of highest aggregation observed within the surveyed cave, indicating a spatially concentrated population patch typical of the species’ clumped distribution pattern. The size distribution of the *Pinna rudis* population was determined using maximum shell width (Wmax) measurements obtained directly from the 3D photogrammetric model of the 31 identified individuals (Figure 4a). The average for Wmax recorded was 15.2 cm with the most frequent size classes being 10–15 cm and 15–20 cm, comprising 15 and 14 individuals, respectively. Together, these size ranges represent over 90% of the total population. These results describe a size-frequency distribution in which large individuals predominate, consistent with the partial burial pattern typical of the species. These values closely matched the in situ measurements obtained by divers, in which the same specimens yielded an average maximum width of 15.2 cm, validating the accuracy of the photogrammetric workflow.

Moreover, in addition to width, the maximum exposed height (Hexp)—the distance between the upper visible point of the shell and the sediment surface— was derived from the 3D models to better describe the individual structure. The average exposed height of the individuals was 10.8 cm, representing roughly 71% of the total shell width, a proportion consistent with partial burial observed in this species.

### 3.3. Validation of 3D-Derived Measurements

Maximum shell width values obtained from 3D photogrammetric models closely matched those measured in situ by divers (Figure 4b). Mean Wmax derived from 3D models was 15.19 ± 3.23 cm (range: 9.88–21.97 cm), while in situ measurements averaged 15.35 ± 3.48 cm (range: 9.30–21.90 cm). The mean difference between the two methods was −0.16 ± 0.82 cm (95% CI: −0.46 to 0.14 cm), indicating a negligible systematic bias (Figure 4c). The mean absolute error was 0.65 cm, corresponding to an average relative error of 4.34%. The root mean square error (RMSE) was 0.82 cm, and 93.5% of individual measurements (29 out of 31) fell within ±1 cm of the in situ reference value. Pearson correlation analysis revealed a strong linear relationship between both measurement techniques (r = 0.97). Linear regression of 3D-derived on in situ measurements yielded a slope of 0.90 (95% CI: 0.82–0.99) and an intercept of 1.32 cm (95% CI: 0.04–2.59), with R^2^ = 0.95. Bland–Altman analysis confirmed a high level of agreement, with 95% limits of agreement ranging from −1.76 to 1.44 cm (95% CI of upper LoA: 0.93 to 1.96 cm; 95% CI of lower LoA: −2.28 to −1.24 cm). Prior to Bland–Altman analysis, the normality of differences was assessed using the Shapiro–Wilk test (W = 0.917, *p* = 0.020). Although a mild departure from normality was detected, attributable to two individuals (IDs 7 and 16) with discrepancies exceeding 2 cm, the Wilcoxon signed-rank test confirmed that the systematic bias was not significantly different from zero (*p* = 0.468). The two outlying measurements likely reflect local visibility constraints or suboptimal specimen orientation within the photogrammetric model. No size-dependent bias was observed, and most measurements fell well within the expected error range.

## 4. Discussion

This work integrates high-resolution imaging and computational modelling into the framework of marine conservation, providing empirical evidence that supports the use of SfM photogrammetry as a robust, repeatable, and scalable tool for biodiversity assessment in protected marine areas. Ultimately, it contributes to the development of a validated methodological framework for monitoring Mediterranean benthic assemblages and for informing conservation strategies aimed at preserving the structural and functional integrity of these ecosystems.

In the marine environment, seabeds with a high degree of three-dimensional complexity and intricate structures are often associated with high biodiversity. These habitats can be characterized either by geomorphologically complex substrates, such as steep rocky bottoms, or by biogenic structures formed by habitat-building species that create highly heterogeneous environments. For effective management of these complex seabeds, low-resolution spatial techniques such as acoustic mapping or broad-scale visual transects are insufficient; instead, high-resolution data acquisition methods are required. In this context, underwater SfM photogrammetry has emerged as a powerful tool to capture fine-scale topographic and structural features of benthic habitats, enabling the derivation of ecologically meaningful metrics such as rugosity, surface complexity, and microhabitat variability [2,4,32]. These high-resolution 3D reconstructions have proven particularly valuable in rocky and biogenic habitats, where traditional survey techniques often fail to adequately resolve the spatial heterogeneity that underpins biodiversity patterns.

Population metrics, size structure, and morphometric parameters, which are difficult to measure accurately in situ due to time constraints, limited visibility, and the fragility of the species, can be efficiently extracted from 3D photogrammetric reconstructions. The ability to measure variables such as exposed height expands the range of descriptors available for morphometric and ecological analysis, enabling more precise assessments of individual condition, sedimentation level, and potential burial dynamics. Furthermore, the 3D models allow the calculation of composite morphometric indices, such as volume-to-area ratios or shape complexity coefficients, which can be used to characterize shell morphology in specimens with irregular or non-symmetric forms, conditions under which traditional linear measurements are often insufficient. These derived metrics provide valuable tools for refined population assessment and can serve as indicators of environmental conditions, sedimentation, or hydrodynamic stress.

The accuracy levels achieved in the present study are consistent with those reported for other vulnerable benthic taxa monitored through SfM photogrammetry in Mediterranean and temperate environments. Photogrammetric surveys conducted in semi-submerged marine caves have enabled the quantification of abundance and spatial distribution of sessile benthic assemblages, confirming that SfM can deliver robust ecological data even in confined, low-light environments comparable to those encountered in the Foradada Siphon cave [6,33]. Similarly, applications to other structurally complex and protected organisms -including gorgonian forests, cold-water corals, and calcareous sponges- have demonstrated that SfM-derived 3D models provide centimetre-scale morphometric precision suitable for population-level analyses [22,23,25,34]. Taken together, these precedents reinforce the transferability of our methodological approach to other protected bivalve and invertebrate species across the Mediterranean basin, particularly in the context of MPAs where minimally invasive techniques are a prerequisite. A direct quantitative comparison with previous SfM-based morphometric studies highlights the competitive performance of our workflow. Palma et al. [22] reported mean errors of approximately 1.0 cm for *Paramuricea clavata* colonies; Prado et al. [23] documented errors of 1–2 cm for gorgonian colonies in Le Danois Bank; and Price et al. [25] reported subcentimetre to centimetre-scale precision for cold-water coral reefs. The mean absolute error obtained here (0.65 cm) and the relative error of 4.34% therefore fall within or below the upper end of the precision range reported for comparable SfM applications, while extending the methodology to a previously untested target taxon (Pinnidae) and to particularly demanding acquisition conditions (semi-confined, low-light cave habitat). This combination of validated metric accuracy and adaptation to challenging environments distinguishes the present contribution from previous studies, which have largely focused on rocky reef or open-water sessile assemblages.

The relevance of morphometric data for assessing *Pinna rudis* populations has been highlighted by recent studies, including field-based size measurements across expanding distribution ranges [21]. The use of SfM photogrammetry facilitated precise identification and measurement of individuals, overcoming limitations associated with traditional visual census methods. Furthermore, the high-resolution models provide a baseline for long-term monitoring, enabling detection of potential population declines due to environmental stressors or anthropogenic disturbances. The application of SfM photogrammetry in marine conservation has proven to be a powerful tool for non-invasive monitoring of benthic species such as *P. rudis*. The findings from the Cabrera Archipelago highlight the effectiveness of this approach in mapping distribution patterns and tracking population changes over time. Continued use of photogrammetry, coupled with conservation measures, will be essential for ensuring the protection and sustainability of *Pinna rudis* populations in Mediterranean marine protected areas. Several characteristics of the present workflow indicate that it is well suited for long-term monitoring schemes. First, the georeferenced 3D models constitute permanent, archivable digital baselines that can be re-examined years later by independent observers, ensuring methodological reproducibility across temporal cohorts of researchers. Second, the modular design of the photogrammetric system allows for unchanged optical and illumination configurations across successive surveys, minimising methodological drift. Third, by combining individual-level identification (specimen labels) with metric scaling, the same individuals can be tracked over time, enabling the estimation of demographic parameters such as growth rates, recruitment and mortality with the same models. Fourth, the non-invasive nature of the technique reduces cumulative disturbance over repeated visits, a critical consideration in MPAs hosting protected species. These features collectively position SfM photogrammetry as a robust complement -rather than a one-off alternative- to existing diver-based demographic monitoring programs.

It is important to highlight that the low measurement error (<1 cm) and the strong agreement with in situ data demonstrate that SfM-derived 3D models provide a reliable and non-invasive alternative for morphometric monitoring of *Pinna rudis*. Importantly, the photogrammetric approach enables the extraction of additional parameters such as exposed height/surface, which are difficult to measure during traditional dive-based surveys due to time constraints, depth limitations, and the protected status of the species. These results highlight the potential of 3D photogrammetry not only to replicate conventional measurements with high accuracy, but also to expand the range of morphometric descriptors available for population and ecological studies.

Despite the strong performance of the photogrammetric workflow, certain technical limitations inherent to underwater SfM deserve consideration. The absence of GNSS metadata in the image dataset required reliance on manual tie points and specimen identification labels as the sole source of metric scaling. In addition, the presence of mobile elements within the field of view—such as macroalgae, seagrass canopies, or suspended fauna—can introduce motion blur and geometric distortions in the reconstructed models, representing a relevant limitation for the application of this methodology in vegetated or hydrodynamically active habitats. While this approach proved effective -yielding sub-centimetre accuracy with a mean scale error of ±0.001 m it introduces dependency on the regular placement and visibility of reference targets throughout the survey area. Future implementations could benefit from the integration of stereo-camera systems or inertial navigation units directly coupled to the imaging platform, which would reduce reliance on in-scene scale references and facilitate georeferencing without ground control points. Recent comparative assessments of diver-operated and ROV-mounted photogrammetry systems have shown that both approaches achieve camera alignment rates above 90%, though diver-derived surveys tend to produce higher point cloud densities, particularly for fine-scale morphological analysis in smaller survey areas [35]. These findings suggest that diver-operated systems, such as the custom module developed in this study, remain particularly well suited for detailed specimen-level measurements, while ROV-based approaches may be more efficient for surveying larger spatial extents. Water visibility and turbidity also constitute key environmental constraints for the application of underwater SfM photogrammetry. In the present study, the Foradada Siphon cave offered favourable acquisition conditions, with horizontal visibility consistently exceeding 10 m and minimal suspended particulate matter, factors that contributed decisively to the high calibration rate (99% of frames aligned) and to the geometric accuracy of the reconstruction. Lower visibility, frequently encountered in coastal or sediment-rich settings, is known to degrade keypoint detection and feature matching, resulting in incomplete models or systematic distortions [36,37]. Likewise, suspended particles, biological aggregates, and backscatter from artificial illumination can compromise both image contrast and apparent geometry, particularly when the camera-to-substrate distance increases beyond ~2 m. Mitigation strategies include the use of broad-spectrum continuous lighting positioned away from the optical axis to reduce backscatter as implemented in the custom module developed here shorter camera-to-subject distances, slower diver speeds and image acquisition during periods of calm hydrodynamic conditions. The transferability of the workflow to more turbid Mediterranean settings (e.g., shallow *Posidonia oceanica* meadows or mixed substrates near river mouths) will therefore require dedicated assessment.

From a conservation management perspective, the methodology validated here offers significant practical advantages for the long-term monitoring of *Pinna rudis* populations within marine protected areas. The 3D models generated in this study not only provide a spatial baseline for demographic comparisons across annual campaigns but also capture habitat-level information—substrate texture, sediment depth, and microhabitat heterogeneity, presence of invasive species—that can inform assessments of habitat suitability and degradation. Under conditions comparable to those of the present study—confined, low-light environments with legally protected species where physical contact and repeated diver disturbance must be minimised—photogrammetric approaches can require significantly less field time and diver disturbance than traditional quadrat-based methods, while providing equivalent or richer spatial and morphometric data [2,4]. In the context of species under legal protection, such as *P. rudis* under the Barcelona Convention, the reduction in diver disturbance time is not only a logistical advantage but also a regulatory one. Beyond the present pilot validation, the standardized, repeatable nature of SfM-derived datasets offers a foundation for the future development of shared monitoring protocols across MPAs in the Mediterranean. Such protocols could, in turn, facilitate regional-scale comparative analyses of population status, particularly in the context of the documented range expansion of the species across the Mediterranean basin [11,38].

Looking ahead, the integration of SfM photogrammetry with emerging technologies opens promising possibilities for marine biodiversity monitoring. The photogrammetric module developed in this study was conceived from the outset with ROV integration capability, and the deployment of such systems on autonomous platforms would substantially reduce the physical and logistical constraints associated with scuba diving, particularly at depths beyond 30 m, where bottom time is severely limited. The combination of 3D photogrammetry with convolutional neural network-based taxonomic identification has demonstrated the capacity to deliver accurate estimates of benthic species abundance and morphology across morphologically complex taxa, and can be applied across SCUBA, ROV, and AUV survey platforms, making it particularly valuable for spatially extensive surveys where manual analysis is prohibitively time-consuming [39]. Applied to *P. rudis*, such workflows could enable semi-automated detection, segmentation, and measurement of individuals directly from 3D point clouds or orthomosaics, dramatically accelerating data processing. Furthermore, the establishment of georeferenced 3D models as annual baselines will allow future studies to quantify population changes at the individual level (tracking recruitment, growth, and mortality), thereby providing the demographic resolution needed to evaluate the conservation status of the species under conditions of environmental change.

## 5. Conclusions

Although SfM photogrammetry has been increasingly applied to benthic habitats, quantitative validations of its metric accuracy for protected marine species remain limited, particularly in confined and low-light environments such as marine caves. Here, we provide one of the few validations of SfM-derived morphometric measurements for a legally protected bivalve, *Pinna rudis*, through direct comparison with in situ diver measurements. By integrating a custom low-light photogrammetry system with an ecological validation framework, this study offers a promising approach with potential for broader application for the non-invasive monitoring of vulnerable benthic species in marine protected areas. From a conservation standpoint, these findings have potential practical implications. The validated workflow has the potential to be incorporated into existing demographic monitoring programs of *Pinna rudis* in the Cabrera Archipelago and other Mediterranean MPAs, potentially providing managers with quantitative tools to detect changes in density, size structure and habitat condition without compromising the integrity of the populations. Given the species’ protection under the Barcelona Convention and its role as a potential ecological replacement for the critically endangered *P. nobilis*, the establishment of standardised, repeatable and minimally invasive monitoring protocols is a conservation priority. The methodology presented here directly contributes to that need, supporting evidence-based decision-making in the management of vulnerable benthic communities and the preservation of the structural and functional integrity of Mediterranean marine ecosystems.

## Figures and Tables

**Table 1:** Summary of model performance.

Parameter	Value	Unit/Description
Number of acquired images	1217	Total frames captured
Calibrated images	1209 (99%)	Successfully aligned
Mean reprojection error	0.477	Pixels
Total 3D keypoints	10,068,786	After triangulation
Dense point cloud	367,801,416	Total reconstructed points
Area covered	86.1	m^2^
Scale bar accuracy	±0.001	m (mean scale error −0.0 m)
Camera optimization difference	3.02%	Between initial and optimized parameters
Average matched keypoints	33,111	Per image
Point cloud density	High	Full-resolution reconstruction

## Data Availability

Affiliated institutions have a data sharing protocol; therefore, raw or processed data will be made available on request.

## Abbreviations

The following abbreviations are used in this manuscript:

SfM	Structure from Motion
DSM	Digital Surface Model
MPAs	Marine Protected Areas
CAMTNP	Cabrera Archipelago Maritime-Terrestrial National Park
ROV	Remotely operated vehicle
POM	Polyoxymethylene
LED	Light-Emitting Diode
Wmax	Maximum shell width
Hexp	Maximum exposed height
